# Architectural diversity and galling insects on *Caryocar brasiliense* trees

**DOI:** 10.1038/s41598-017-16954-6

**Published:** 2017-11-30

**Authors:** Germano Leão Demolin Leite, Ronnie Von dos Santos Veloso, José Cola Zanuncio, Alcinei Mistico Azevedo, Júlia Letícia Silva, Carlos Frederico Wilcken, Marcus Alvarenga Soares

**Affiliations:** 1 Insetário G.W.G. Moraes, Instituto de Ciências Agrárias, Universidade Federal de Minas Gerais, 39.404-547 Montes Claros, Minas Gerais Brazil; 2 Departamento de Entomologia/BIOAGRO, Universidade Federal de Viçosa, 36570-900 Viçosa, Minas Gerais Brazil; 3 Programa de Pós-Graduação em Produção Vegetal, Universidade Federal dos Vales do Jequitinhonha e Mucuri, 39100-000 Diamantina, Minas Gerais Brazil; 4 Departamento de Proteção Vegetal, Faculdade de Ciências Agronômicas, Universidade Estadual Paulista “Júlio de Mesquita Filho”, Fazenda Lageado, CEP 18610-307 Botucatu, São Paulo Brazil

## Abstract

Galling insects are a highly sophisticated herbivore group on *Caryocar brasiliense*, a tree that represents the main income source for many communities. The effect of architectural diversity of *C. brasiliense* trees on galling insect community diversity and abundance was studied. The abundance of adult insects and galled leaves were seven and 1.6 times higher in trees with a greater height/width of canopy (RHW) ratio, respectively. Gall parasitoid richness was 1.8 times greater on trees with higher RHW. *Zelus armillatus* (Lepeletier & Serville) (Hemiptera: Reduviidae) and ant numbers were 5.8 and 2.7 higher on trees with the largest and smallest RHW, respectively. More complex plant architectures favored species diversity for galling insects and their natural enemies. The competition among four galling insect species for space and feeding and the evidence of “prudence strategy” were, for the first time, observed for galling insects in the Brazilian Cerrado biome.

## Introduction

Galling insects are a highly sophisticated herbivore group^1^ and many of them damage economically important plants^2^. They developed the ability to modify host plant tissue to produce specialized structures where their larvae could develop protected from harsh environmental conditions while feeding on a rich food source^3,4^.

Changes to the host plant affect interactions with galling insects. The diversity and abundance of these insects differ with higher species numbers on more architecturally complex hosts^5–7^. On the other hand, the factors affecting the galling community and population trends of galling insects need to be studied at a local scale^8^ especially given that host traits mediated habitat interactions.

A system with *Caryocar brasiliense* Camb. (Malpighiales: Caryocaraceae), common and economic cerrado (savanna) tree, and four galling herbivores in central Brazil is appropriate to evaluate the hypotheses presented^9,10^. Galls induced by four hymenopteran species are distinct in shape and morphology, and abundant^11–14^, facilitating data collection and testing of hypotheses. The canopy of a tree is a small-scale biogeographic island and an example to test this hypothesis^15^. A more aggressive galling insect species could affect a member of this group or even extinguish it by altering the environment, as observed for exotic versus native plants^16^. Biogeographic island predicts that extinction rates are higher on smaller islands because they cannot withstand high populations of organisms with the rarest species being vulnerable to extinction^16^. Biogeographic island involves the history of the biological processes such as colonization, speciation and extinction to explain species distribution patterns^17^. Smaller trees would provide smaller rather than larger islands.

*Caryocar brasiliense* trees are protected by Brazilian federal law and represent the main income source of many communities^10^. These trees remain in Cerrado lands transformed into pasture or agricultural land in a common scenario of isolated individuals in the agro-urban-landscape. The effects of host plant attributes on the diversity and abundance of galling insects and their natural enemies on *C. brasiliense* trees were evaluated in a pasture area. The hypotheses that more complex host individuals – larger trees - (i.e. biogeographic island) support a higher diversity of galling insects and their community was tested^7,18^. Therefore, we expect to find more species and individuals of galling insects, parasitoids and predators on larger trees.

## Results

### Architectural diversity and galling insects

In comparison with trees with a smaller RHW, *Caryocar brasiliense* trees with a greater height/width canopy ratio (RHW) had 7.0 times the number of galling insects, 7.8 times more *Eurytoma* sp. (globoid galls) adults, 1.6 times the percentage of galled leaves, 7.2 times greater leaflet area with galls, 2.2 times more leaf area (mm^2^) occupied by *Eurytoma* globoid galls, and 3.9 times the number of *Eurytoma* globoid galls. On the other hand, the number of Hymenopteran discoid galls was 7.8 times higher on *C. brasiliense* trees with lower RHW than on those with greater RHW.

**Figure 1:**
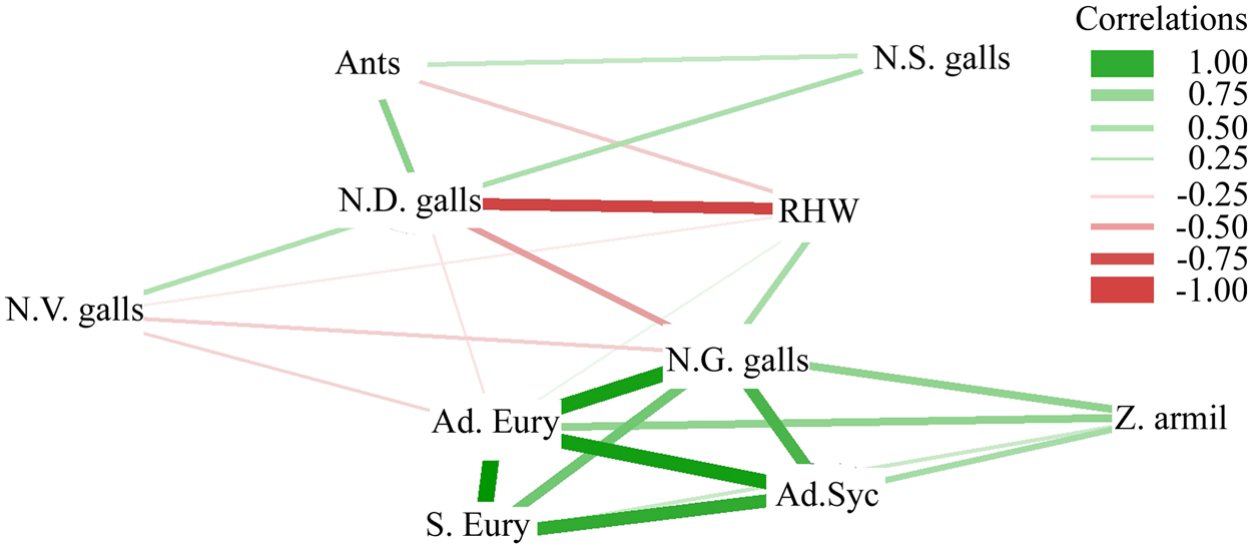
Estimated network structures based on Spearman correlation (*P* < 0.05) generated for globoid galls (N.G. galls), vein galls (N.V. galls), spherical galls (N.S. galls) and discoid galls (N.D. galls), *Eurytoma* sp. (Ad. Eury) adults and their survivals (%) (S. Eury), *Sycophila* sp. (Ad. Syc), *Zelus armillatus* adults (Z. Armil) and ants per *Caryocar brasiliense* tree leaflet and height/width of canopy ratio (RHW) characteristics. Montes Claros, Brazil, autumn 2013 to autumn 2016.

The *C. brasiliense* RHW did not affect diversity and richness of the galling insect, abundance of adult *Bruchophagus* sp. vein galls, Eulophidae spherical galls, or Hymenopteran discoid galls, the leaf area (mm^2^) occupied by *Bruchophagus* sp. vein galls, Eulophid spherical galls and hymenopteran discoid galls, width (mm) of conglomerate of globoid galls, gall numbers of *Bruchophagus* sp. vein galls and Euplophid spherical galls (Tables 1–3). The effect of *C. brasiliense* RHW on adult survival rates for *Eurytoma* globoid galls was not significant but they had a survival rate 2.6 times higher on higher RHW trees. The increase in the number of adults of *Eurytoma* sp. globoid galls and their galls reduced the number of Hymenopteran discoid galls and *Bruchophagus* sp. vein galls (Fig. 1).

**Table 1:** Ecological indices of galling insects and their natural enemies per *Caryocar brasiliense* leaflet as function of the height/width canopy ratio (RHW). Montes Claros, Brazil, autumn 2013 to autumn 2016.

Ecological indices	Height/width of canopy ratio (RHW)			*ANOVA*	
	RHW 1	RHW 2	RHW 3	*F*	*P*
Gall abundance*	0.39 ± 0.28b	0.41 ± 0.16b	2.94 ± 1.47a	4.862	0.01784
Gall richness^n.s.^	0.83 ± 0.16a	1.25 ± 0.19a	0.92 ± 0.08a	1.571	0.23019
Gall diversity ^n.s.^	1.15 ± 0.21a	1.69 ± 0.21a	1.20 ± 0.16a	2.875	0.07776
Parasitoid abundance^n.s.^	0.10 ± 0.05a	0.05 ± 0.02a	0.28 ± 0.11a	2.562	0.09996
Parasitoid richness**	1.00 ± 0.21b	0.67 ± 0.22b	1.50 ± 0.26a	3.732	0.04022
Parasitoid diversity ^n.s.^	1.16 ± 0.21a	1.00 ± 0.34a	1.57 ± 0.21a	2.052	0.15240
Predator abundance ^n.s.^	0.56 ± 0.14a	0.45 ± 0.15a	0.40 ± 0.14a	0.332	***
Predator richness ^n.s.^	3.50 ± 0.66a	2.92 ± 0.48a	3.25 ± 0.30a	0.414	***
Predator diversity ^n.s.^	5.93 ± 1.39a	5.32 ± 1.22a	5.62 ± 0.76a	0.091	***

Means (±standard error) followed by the same letter, per row, are similar by the test of Scott-Knott (*P < 0.01 and **P < 0.05). Values of *F* and *P* were obtained by ANOVA. n.s. = non-significant by ANOVA. ***highly non-significant. The dfs of treatments, blocks, and errors were 2, 11, and 22, respectively. N = 36. The categories were: RHW 1: 1.00 to 1.64, RHW 2: 1.64 to 2.10 and RHW 3: 2.11 to 2.53 m.

**Table 2:** Number of adult galling insects and their natural enemies and of *Eurytoma* sp., *Sycophila* sp., *Ablerus magistretti* and *Quadrastichus* sp. survival (%) per *Caryocar brasiliense* leaflet as function of height/width canopy ratio (RHW). Montes Claros, Brazil, autumn 2013 to autumn 2016.

Kinds of insects	Height/width of canopy ratio (RHW)			*ANOVA*	
	RHW 1	RHW 2	RHW 3	*F*	*P*
**Galling insects**					
*Eurytoma* sp.*	0.39 ± 0.28b	0.36 ± 0.16b	2.94 ± 1.47a	5.179	0.01435
*Bruchophagu* sp.^n.s.^	0.00 ± 0.00a	0.02 ± 0.01a	0.00 ± 0.00a	1.000	***
Eulophidae ^n.s.^	0.00 ± 0.00a	0.003 ± 0.001a	0.00 ± 0.00a	1.941	0.16734
Hymenoptera ^n.s.^	0.001 ± 0.001a	0.052 ± 0.050a	0.00 ± 0.00a	1.018	0.37785
**Natural enemies**					
*Ablerus magistretti* ^n.s.^	0.00 ± 0.00a	0.00 ± 0.00a	0.01 ± 0.01a	2.570	0.09929
*Epipolops* sp.^n.s.^	0.03 ± 0.01a	0.03 ± 0.01a	0.00 ± 0.00a	4.258	0.05734
*Holopothrips* sp.^n.s.^	0.04 ± 0.01a	0.01 ± 0.01a	0.04 ± 0.01a	2.355	0.11835
*Quadrastichus* sp.^n.s.^	0.01 ± 0.01a	0.01 ± 0.01a	0.01 ± 0.01a	0.217	***
Spiders^n.s.^	0.04 ± 0.01a	0.02 ± 0.01a	0.06 ± 0.03a	0.778	***
*Sycophila* sp.^n.s.^	0.10 ± 0.05a	0.05 ± 0.01a	0.27 ± 0.11a	2.716	0.08830
Total ants**	0.31 ± 0.09a	0.17 ± 0.04b	0.06 ± 0.01b	4.670	0.02039
*Trybonia* spp.^n.s.^	0.11 ± 0.05a	0.21 ± 0.16a	0.01 ± 0.01a	1.061	0.36320
Zelus armillatus**	0.03 ± 0.01b	0.01 ± 0.01b	0.23 ± 0.10a	3.716	0.04072
**Survival (%)**					
*Eurytoma* sp.^n.s.^	7.16 ± 4.24a	6.34 ± 2.15a	17.47 ± 6.64a	1.873	0.17732
*Sycophila* sp.^n.s.^	25.67 ± 9.06a	12.08 ± 5.63a	9.49 ± 1.22a	1.941	0.16730
*Ablerus magistretti* ^n.s.^	0.00 ± 0.00a	0.00 ± 0.00a	0.44 ± 0.36a	1.505	0.24407
*Quadrastichus* sp.^n.s.^	2.16 ± 1.41a	4.88 ± 3.40a	1.93 ± 1.12a	0.755	***

Means (±standard error) followed by the same letter, per row, are similar by the test of Scott-Knott (*P < 0.01 and **P < 0.05). Values of *F* and *P* were obtained by ANOVA. n.s. = non-significant by ANOVA. ***highly non-significant. The dfs of treatments, blocks, and errors were 2, 11, and 22, respectively. N = 36. The categories were: RHW 1: 1.00 to 1.64, RHW 2: 1.64 to 2.10 and RHW 3: 2.11 to 2.53 m.

**Table 3:** Damage types, percentage of galled leaflet (Leaflet%), leaflet area of all galls (Galls%), *Eurytoma* globoid galls (mm^2^) (E.g. galls), *Bruchophagus* vein galls (mm^2^) (B. v. galls), Euplophid spherical galls (mm^2^) (E.s. galls), hymenopteran discoid galls (mm^2^) (Disc. galls), length (mm) of conglomerate globoid galls (C. glob. galls), width (mm) of conglomerate globoid galls (W.C.G.Gs.), numbers of globoid galls (N.G. galls), vein galls (N.V. galls), spherical galls (N.S. galls) and discoid galls (N.D. galls) per leaflet of *Caryocar brasiliense* trees as function of height/width of canopy ratio (RHW). Montes Claros, Brazil, autumn 2013 to autumn 2016.

Damage	Height/width of canopy ratio (RHW)			*ANOVA*	
	RHW 1	RHW 2	RHW 3	*F*	*P*
Leaflet%**	31.55 ± 4.34b	30.80 ± 5.64b	50.16 ± 5.84a	3.798	0.03830
Galls% *	2.38 ± 0.64b	2.14 ± 0.59b	16.22 ± 3.75a	13.223	0.00017
E.G. galls*	170.27 ± 28.87b	152.78 ± 26.68b	360.38 ± 72.74a	5.540	0.01126
*B.v*. galls ^n.s.^	11.11 ± 1.21a	11.75 ± 1.80a	0.00 ± 0.00a	0.768	***
(E.s. galls)^n.s.^	1.64 ± 0.83a	2.21 ± 0.92a	0.79 ± 0.01a	0.734	***
(D. galls)^n.s.^	3.69 ± 0.44a	2.46 ± 2.97a	4.12 ± 0.01a	1.495	0.29727
L.C.G.G**	19.80 ± 2.23b	18.96 ± 1.82b	28.53 ± 3.21a	3.734	0.04016
W.C.G.G.^n.s.^	5.92 ± 0.55a	5.62 ± 0.57a	7.98 ± 0.86a	3.372	0.05281
N.G. galls*	3.45 ± 0.67b	3.66 ± 0.75b	13.85 ± 2.20a	18.500	0.00002
N.V.galls ^n.s.^	0.15 ± 0.08a	0.03 ± 0.02a	0.00 ± 0.00a	2.559	0.10022
N.S. galls ^n.s.^	0.18 ± 0.09a	0.03 ± 0.02a	0.02 ± 0.01a	2.388	0.11518
N.D. galls*	0.97 ± 0.21a	0.19 ± 0.09b	0.06 ± 0.05b	11.616	0.00036

Means (±standard error) followed by the same letter, per row, are similar by the test of Scott-Knott (*P < 0.01 and **P < 0.05). Values of *F* and *P* were obtained by ANOVA. n.s. = non-significant by ANOVA. ***highly non-significant. The dfs of treatments, blocks, and errors were 2, 11, and 22, respectively. N = 36. The categories were: RHW 1: 1.00 to 1.64, RHW 2: 1.64 to 2.10 and RHW 3: 2.11 to 2.53 m.

### Architectural diversity and natural enemies

Species richness of gall parasitoids was 1.8 times higher on *Caryocar brasiliense* trees with a higher RHW. The *C. brasiliense* RHW did not affect abundance or diversity of gall parasitoid species, but their values were 1.8 and 1.4 times greater, respectively, on higher RHW trees. The numbers of *Quadrastichus* sp. (Hymenoptera: Eulophidae) and *Ablerus magistretti* Blanchard (Hymenoptera: Aphelinidae) adults were similar between trees with low and high RHW, but the latter species was observed only on higher RHW trees.

*Caryocar brasiliense* RHW did not affect the number of *Sycophila* sp. (Hymenoptera: Eurytomidae) adults, but this insect had numbers 1.8 times higher on larger RHW trees. The effect of *C. brasiliense* RHW on the percentage of *Sycophila* sp. and *Quadrastichus* sp. adult survival was not observed, but both showed 2.4 times greater survival on lower and intermediate RHW trees, respectively (Tables 1–2).

The abundance, diversity and richness of predator species and the numbers of *Epipolops* sp. (Hemiptera: Geocoridae), *Holopothrips* sp. (Thysanoptera: Phlaeothripidae), spiders and *Trybonia intermedius* Bagnall and *Trybonia mendesi* Moulton (Thysanoptera: Phlaeothripidae) were similar between trees with different RWH. However, *C. brasiliense* trees with larger and smaller RHW had 5.8 and 2.7 times greater *Zelus armillatus* (Lepeletier & Serville) (Hemiptera: Reduviidae) and ant numbers, respectively (Tables 1–2).

Adult numbers and survival (%) of the parasitoid *Sycophila* sp. and the predator *Z. armillatus* increased with the frequency of *Eurytoma* sp. globoid galls and their galls (Fig. 1). On the other hand, the greater number of individuals of the predator *Z. armillatus* reduced the survival (%) of the parasitoid *Sycophila* sp. [Survival of *Sycophila* sp. (%) = 7.61 + 101.32x²√*Z. armillatus* − 104.00 x *Z. armillatus*, F = 3.34, P = 0.0477, R^2^ = 0.17]. Ants positively affected the numbers of Eulophid spherical galls and those of Hymenopteran discoid gall (Fig. 1).

## Discussion

### Architectural diversity and galling insects

The attacks more frequently of galling insects for taller *C. brasiliense* trees or those with wider crowns confirms the hypothesis that larger trees support more species and individuals of galling insects and their communities (i.e. parasitoids and predators)^7,18^ and the prediction of the plant vigour hypothesis (PVH). The PVH indicates oviposition preference of females and higher offspring performance of herbivorous insects on fast-growing plants (plant modules). Sawfly galls attacked more frequently longer shoots without galls and those with two, three, four, or five galls were successively longer than with fewer galls^19^. Bigger plants typically support more galling insect species^5,20^.

Galling insect preference for *C. brasiliense* trees in relation to height and canopy width are of two groups: i) *Eurytoma* sp. (most abundant) and ii) other three galling insect species. *Eurytoma* (globoid galls) preferred taller trees (RHW 3) where it showed higher gall numbers and area and consequently a higher percentages of galled leaflets and greater area with galls. The second group, Eulophidae spherical galls, Hymenopteran discoid galls, and *Bruchophagus* sp. vein galls did not prefer taller trees, but those with wider crowns (i.e. Hymenopteran discoid galls) generally showed higher numbers of its galls. *Bruchophagus* vein galls were not found on taller trees (RHW 3). The question why *Eurytoma* sp. attacks more frequently taller *C. brasiliense* trees and the other three galling insects a wider canopy may be explained by the fact that leaves on the top of taller *C. brasiliense* trees probably suffer a drying effect from the wind that shorter trees do not especially in regions with high temperatures and sunlight and low relative humidity^10^. Higher fruit production in the basal part than in the apical part of *C. brasiliense* canopy increased flower and fruit drop by winds^10^. This indicates that leaves more exposed to sunlight and winds can negatively affect their resistance to galling insects^21^. The higher wind and sunlight incidence harden leaves and may also explain the higher population of *Eurytoma* sp. on taller *C. brasiliense* trees. These leaves are, probably, a better food source for galling insects and *Eurytoma* sp. seems to predominate on them. Harsh ecophysiological conditions in the upper canopy of tropical rainforests increase gall-forming populations. Sclerophyll leaves increased with tree height while free-feeding herbivores decreased inversely^22^. Water and nutrient stress in the canopy tree meristem in tall wet tropical rainforests may cause leaf sclerophylly, forming a suitable ecosystem for gall-forming insects^22^. The other three galling insect species also prefer leaves more exposed to wind and sun^12–14^, but they preferred trees with a wider canopy than taller trees, avoiding competition with *Eurytoma* sp.

The pattern changes of the three galling insect species between *C. brasiliense* trees with low or high *Eurytoma* sp. density is related to sunlight and wind exposure, gall distribution on the leaf (i.e. border/interior of the plant canopy, border/near the mid vein, distal/proximal-near to petiole), and branch level (i.e. north/south)^21^. Most galling insects preferred the *C. brasiliense* leaves more exposed to the wind and sunshine, on the branch and at the leaf border and median parts^11–14^. However, other galling insects attacked other parts of *C. brasiliense* leaves in the presence of high *Eurytoma* sp. populations^21^. This suggests competition between the three galling insect species and *Eurytoma* sp. with the latter showing faster colonization of plants with greater biotic potential^21^. *Caryocar brasiliense* loses its leaves in Aug/Sep with new leaves appearing at the end of September, a period without rainfall and with strong wind and high sunlight^10^. *Eurytoma* sp. induces galls on young expanding C*. brasiliense* leaves when the wasp females were easily found ovipositing on unfolded leaves. Approximately two days later the oviposition site became reddish with a gall visible a few days later^11^. Plant phenology and colonization periods on *C. brasiliense* leaves explains the decrease of *Eurytoma* gall abundance as the numbers of other galling insects increased throughout the year as well as the differential temporal distribution of galling insects^23^. Chemical or visual markers may indicate which galling insect dominates a particular ecological niche (i.e., part of a leaf, branch or even a tree)^21^. The genetic differences between *C. brasiliense* plants and the chemical or morphological composition with different leaf parts, branch positions, sun/wind exposure or relationships with other arthropods (i.e., natural enemies)^21^ may also be responsible for this process or these differences.

The reduction of the galling insect communities on *C. brasiliense*, which acts as a biogeographic islands for these insects, can be explained by the collect of fruits without control affecting plant propagation. *Caryocar brasiliense* had only about 7.96 and 10.65% of individuals up to 1.0 m tall and 59.58 and 44.73% above 3.0 m (reproductive phase) in the cerrado and pasture areas, respectively^10^. This indicates that collectors remove nearly all fruits from the tree thus reduce *C. brasiliense* propagation in the cerrado of Brazil. *Eurytoma* sp. attack more frequently larger trees and the other three species of insect galling the smaller trees, which increases the risk of their extinction due to the inadequate renewal of *C. brasiliense* trees. This agrees with reports for exotic plants that can extinguish those native to smaller biogeographic islands^16^ and to Lepidoptera communities (i.e. generalists x specialists) in urban parks being affected by their size and human disturbance^15^.

### Architectural diversity and natural enemies

A higher diversity of natural enemies, principally parasitoids, as was found with galling insects in the more complex host individuals, indicates that their populations depend on their prey and host and that they follow of the herbivorous insects^24,25^.

The natural enemies found can be divided into parasitoids and predators. The first group (i.e. *Sycophila* sp. and *A. magistretti*) showed the same trend as its hosts (galling insects) with higher richness, diversity and abundance on wider crown trees. *Sycophila* sp., a major *Eurytoma* sp.^23,26^ parasitoid showed a higher survival rate on lower RHW trees and *Eurytoma* had better fitness on higher RHW trees. The larger RHW trees had the highest number of *Eurytoma* sp. and *Z. armillatus* predators^11,26^. These facts showed an evidence of “prudence strategy”, whereby predators fed on parasitized prey, preserving the healthy prey as a food reserve for future generations, without endangering prey populations^27^. The predator *Z. armillatus* may prefer attacking *Eurytoma* sp. galls parasitized by *Sycophila* sp. The “prudence strategy” has been observed for *Protonectarina sylveirae* (Saussure) (Hymenoptera: Vespidae) with the leafminer *Liriomyza* sp (Diptera: Agromyzidae) and parasitized aphids (Hemiptera: Aphididae)^28^, but this is the first time that it is seen, evidenced, in galling insects. However, a prudence strategy would have to be demonstrated experimentally in future study. Positive relationships between indole butyric acid concentrations and successfully induced globoid galls as well as between the number of adults of the galling *Eurytoma* sp. and its parasitoid *Sycophila* sp. were found^26^. Selecting an enemy-free space could be the main reason why sawfly oviposition patterns with higher values on lower quality plants decreases predation and parasitism^8,29–31^.

The similar abundance, richness and diversity of the second group on *C. brasiliense* with different RHW is due to the fact that they are generalists with little dependence on a single prey, contrary to most parasitoids^32^ and these trees support greater diversity of free-feeding herbivore insects^33–36^. The higher percentages of defoliation and populations of defoliators and ants on *C. brasiliense* trees may be explained by the impact of predatory ants, bugs and spiders reducing defoliators and leaf miner insects on wider crown plants^35^. Variations in abundance and diversity of sucking insects and their natural enemies on wide crown trees and of the number of species and individuals of natural enemies is similar to those for *Dikrella caryocar* (Coelho, Leite & Da-Silva) (Hemiptera: Cicadellidae), of ants, predator thrips and lady beetles as well as with higher values on these *C. brasiliense* trees^36^.

Ants, *Epipolops* sp., *Holopothrips* sp., *Sycophila* sp., spiders, and *Z. armillatus* can be important to control *Eurytoma* sp. on *C. brasiliense* trees^11,23,26,37,38^. The higher numbers of the predator *Z. armillatus* on *C. brasiliense* trees at the University *Campus* might be due to more leaves galled by *Eurytoma* sp. on these trees than in pastureland and cerrado^35^. *Zelus armillatus* preyed on *Eurytoma* galls, which can cover up to 70% of the leaf area^11,26^. These galls can support higher diversity of natural enemies which can kill and cause a top down impact on natural enemies^8,30,31^.

Complexity of plant architecture (i.e. biogeographic island) favored the diversity of galling insect species and their parasitoids and the predator *Z. armillatus*. The patterns of natural enemies indicate that their populations depend on prey/hosts and that they follow those of the herbivorous insects. The competition among the four galling insect species for shelter and feeding space and the evidence of “prudence strategy” were observed for the first time for galling insects.

## Methods

### Study

This study was performed in the municipality of Montes Claros, Minas Gerais state, Brazil over three consecutive years (Jun 2013 through Jun 2016). The region has dry winters and rainy summers, climate Aw: tropical savanna according to Köppen^39^. The study was performed in pasture [S 16°46′16.1″ W 43°57′31.4″at 940 m a.s.l. altitude with dystrophic yellow red latossol soil with loamy texture^34^. The arthropods collected are neither an endangered nor protected species.

The pasture area has 84.2% of the soil covered by grass, 5.8%, 4.8% and 2.8% by herbs, small and tall trees, respectively with an average of 42 *C. brasiliense* trees per ha^34^.

### Host Plants Studied and Galling Species

*Caryocar brasiliense* trees can reach over 10 m tall with a 6 m canopy width^10^. Its fruits have an internal mesocarp rich in oil, vitamins, and proteins with many compounds of medicinal importance. Moreover, it is used by humans for food, production of cosmetics, lubricants, and in the pharmaceutical industry^40–42^.

Adult (reproductive stage) *C. brasiliense* trees in the pasture area were two to nine meters tall with a two to 11 meters width canopy^34^. The hymenopteran galls on *C. brasiliense* leaves are *Bruchophagus* sp. vein galls (Hymenoptera: Eurytomidae), Eulophidae spherical galls (Hymenoptera), *Eurytoma* sp. glodoid galls (Hymenoptera: Eurytomidae) and Hymenopteran discoid galls, the morphology, natural history and tree distribution of which have been described^11–14,21^. Descriptions of the natural enemies and other herbivores in this *C. brasiliense* system were also provided^21^.

### Study Design

The study design was completely randomized with 36 replications (36 trees). Data was collected from *C. brasiliense* adult trees (producing fruits) every 50 m along a 600-m transect. Insect species (i.e., rare species) data was collected over three consecutive years. No fertilizers or pesticides were used.

The distribution of galling insects and their galls, predators, and leaf percentages infested with galls (three leaflets/leaf) were recorded in 12 fully expanded leaves of 36 *C. brasiliense* trees (one leaf per canopy vertical and horizontal stratifications). Sampling was performed monthly in the morning (7–11 AM) by visual observation^43^. Insects were collected with tweezers, brushes, or aspirators and preserved in vials with 70% alcohol for identification by taxonomists. Twelve leaves per tree were collected and transported to the laboratory. Gall size was measured with a digital caliper (accurate to the nearest 0.1 mm). Leaves were scanned and their area and those of each leaf with galling species calculated using a computer program. Subsequently, leaves of each sample were placed in white plastic pots (temperature of 25 °C), and the emergence of galling insects, hyperparasitoids, inquilines and parasitoids evaluated on alternate days for 30 days. The insects emerged were collected and preserved as described for identification by taxonomists. The voucher number for spiders is IBSP 36921–36924 (Instituto Butantan, São Paulo state, Brazil) and that for insects is 1595/02 and 1597/02 (CDZOO, Universidade Federal do Paraná, Paraná state, Brazil).

### Statistical Analyses

Averages were made by reducing the data to leaflet/tree. The richness and species diversity of galling insects and their parasitoids and predators were calculated per tree. The diversity was calculated using Hill´s formula^44^ and the species richness with Simpson indices^45,46^. The height and width (RHW) ratio on *C. brasiliense* trees and percentage survival of galling insect adults [(number of adults/number of galls)*100] and of the parasitoid adults [(number of adults/number of host adults)*100] were calculated. The ratio of height/width categories for *C. brasiliense* trees are: RHW 1: 1.00 to 1.64, RHW 2: 1.64 to 2.10 and RHW 3: 2.11 to 2.53 m with 12 trees per group.

The effect of RHW on ecological indexes and numbers of individuals per herbivore species and natural enemies (transformed to √x + 0.5 or arcsine for percentage data whenever necessary) was tested with ANOVA (*P* < 0.05) and Scott-Knott’s test (*P* < 0.05) and regression analysis were performed with the System of Statistical and Genetics Analysis of the Universidade Federal de Viçosa (UFV)^47^.

The Spearman correlation matrix, among the most significant characteristics, was calculated. The matrices were submitted to correlation networks^48^. The edge thickness was controlled by applying a cut off value of 0.33 (value from which the Spearman correlation became significant, meaning that only edges with $|{{\rm{r}}}_{{\rm{ij}}}|\,\ge 0.33$ were highlighted). All the analyses were performed using the R software version 3.4.1^49^. The correlation network procedure was performed using the package *qgraph* ^48^.
